# The health-care utilization and economic burden in patients with genetic skeletal disorders

**DOI:** 10.1186/s13023-024-03102-3

**Published:** 2024-03-04

**Authors:** Luna Liu, Yingzhou Shi, Xiude Fan, Yangyang Yao, Wanhong Wu, Yang Tian, Huixiao Wu, Zongyue Li, Yanzhou Wang, Chao Xu

**Affiliations:** 1 Department of Endocrinology, Shandong Provincial Hospital, Shandong University, 250021 Jinan, Shandong China; 2 Key Laboratory of Endocrine Glucose & Lipids Metabolism and Brain Aging, Shandong First Medical University, Ministry of Education, Jinan, China; 3 Department of Endocrinology, Shandong Provincial Hospital Affiliated to Shandong First Medical University, 250021 Jinan, Shandong China; 4 Shandong Clinical Research Center of Diabetes and Metabolic Diseases, 250021 Jinan, Shandong China; 5 Shandong Institute of Endocrine and Metabolic Diseases, 250021 Jinan, Shandong China; 6 Shandong Engineering Research Center of Stem Cell and Gene Therapy for Endocrine and Metabolic Diseases, 250021 Jinan, Shandong China; 7 Department of Pediatric Orthopedics, Shandong Provincial Hospital Affiliated to Shandong First Medical University, 250021 Jinan, Shandong China

**Keywords:** Genetic skeletal disorder, Rare bone disease, Health-Care utilization, Economic burden, Health system

## Abstract

**Background:**

Most genetic skeletal disorders (GSD) were complex, disabling and life-threatening without effective diagnostic and treatment methods. However, its impacts on health system have not been well studied. The study aimed to systematically evaluate the health-care utilization and economic burden in GSD patients.

**Methods:**

The patients were derived from 2018 Nationwide Inpatient Sample and Nationwide Readmissions Database. GSD patients were extracted based on International Classification of Diseases-10th revision codes.

**Results:**

A total of 25,945 (0.12%) records regarding GSD were extracted from all 21,400,282 records in NIS database. GSD patients were likely to have significantly longer length of stay (6.50 ± 0.08 vs. 4.63 ± 0.002, *P* < 0.001), higher total charges ($85,180.97 ± 1,239.47 vs. $49,884.26 ± 20.99, *P* < 0.001), suffering more procedure, diagnosis and transferring records in comparison to patients with common conditions. GSD patients had a significantly higher 30-day all-cause readmission rate based on Nationwide Readmissions Database.

**Conclusions:**

The heavy health-care utilization and economic burden emphasized the urgency for policy leaders, scientific and pharmaceutical researchers, health care providers and employers to identify innovative ways and take effective measurements immediately, and eventually to help improve the care, management, and treatment of these devastating diseases.

**Supplementary Information:**

The online version contains supplementary material available at 10.1186/s13023-024-03102-3.

## Introduction

Assessment of the health-care utilization and economic burden of diseases can heighten the awareness to incorporate the provision of adequate clinical services and cost-saving measures for the management of diseases. Previous studies have systematically assessed the prevalence and economic cost of total rare disease or some specific rare disease, such as ectodermal dysplasia, rare orofacial diseases [1–4]. Genetic skeletal disorders (GSD) comprise a diverse range of rare bone conditions characterized by disruptions in skeletal development, growth, and homeostasis [5]. The considerable genetic diversity, clinical variability, and elevated rates of deformities and disability in individuals with GSD underscore the importance of directing attention towards this category of diseases. A small-sample retrospective cohort study has indicated a substantial burden in patients with musculoskeletal diseases, with congenital defects representing only 3.7% of the reported cases [6]. A recent study based on the 2016 Healthcare Cost and Utilization Project (HCUP) databases reported that discharges with rare diseases showed substantially higher charges per discharge and longer length of stay, accounting for nearly half of the US national bill [1]. However, to the best of knowledge, the clinical and economic burden of GSD have not been systematically evaluated.

The latest tenth version of the “*Nosology and Classification of Genetic Skeletal Disorders”* encompasses 461 genetic skeletal disorders under 42 group headings based on their clinical, radiographic, and/or molecular phenotypes, which are associated with mutations in one or more of 437 different genes [7], such as collagen type I alpha 1 chain, phosphate regulating endopeptidase X-linked, pleckstrin homology and RUN domain containing M1, et al. Notably, mutations in different genes can lead to almost identical clinical manifestation and the same gene can result in substantially different clinical phenotypes [8, 9]. In addition to musculoskeletal abnormalities, patients may demonstrate disorders in vision, hearing, cardiac, respiratory, or renal function and have psychological problems [10–14]. The genetic heterogeneity and clinical diversity inherent in GSD necessitate the involvement of a comprehensive and sophisticated clinical care team to establish a definitive diagnosis, then contribute to the prolonged time in diagnosis and treatment, which eventually lead to the under-diagnosis and lack of medical services [8, 15–16]. The absence of effective treatments, limited to symptomatic management can not totally rescue related disease, even accelerate the progression of disease. GSD patients eventually present with high rates of deformity and disability, such as skeletal dysplasia, severe short stature, movement disorders and so on or even death [17–19]. Challenges in diagnosis and the absence of effective treatments typically result in a substantial clinical and economic burden, as well as a poor prognosis for patients with GSD. Therefore, to systematic and comprehensive assess the health-care utilization and economic cost of GSD is a necessary and crucial issue to the public health.

In this study, we explored the health-care utilization and economic burden including total charges, length of stay and early readmission rate in patients with GSD compared to common conditions (CC) based on 2018 HCUP Nationwide Inpatient Sample (NIS) and Nationwide Readmissions Database (NRD). The results showed that patients with GSD or rare bone disease (RBD) possessed heavier health-care utilization than patients with CC in two databases. It indicated the need to increase the health-care assess and adopt cost-saving measures for patients with GSD. These results reminded the policy leaders, health care providers, scientific and pharmaceutical researchers should pay attention to implementing supportive polices, increasing medical care, exploring underlying mechanisms and discovering new drugs for populations with GSD.

## Patients and methods

### Data sources

The NIS is the largest comprehensive, all-payer inpatient database with representative sample and designed for estimates of inpatient health-care utilization, cost, length of stays and outcomes in United States. The NRD is a nationally representative longitudinal database which can be used to track a patient across hospitals via reliable, verified patient linkage numbers within a year and furthermore evaluate the readmission rate. These nationally representative databases are both part of the Healthcare Cost and Utilization Project (HCUP), which was built by the Agency for Healthcare Research and Quality to conduct national medical estimations. Two databases contain both clinical and non-clinical variables to facilitate analysis and incorporate safeguards ensuring the privacy of patients, doctors, and hospitals. The study followed the STROBE reporting guidelines and adhered to the Data Use Agreement of the Healthcare Cost and Utilization Project by the United States Agency for Healthcare Research and Quality. It was exempt from review by the research ethics board, and consent to participate was not applicable. In this study, we utilized NRD database in 2018 (*n* = 12,928,231) and NIS database from 1 January 2016 to 31 December 2018 (*n* = 21,400,282) to evaluate the hospitalized outcomes in patients with GSD compared to patients with common conditions.

### Study population

We distinguished patients with GSD or RBD via using International Classification of Diseases-10th revision (ICD-10) codes in NIS and NRD database. The newest version of the Nosology comprised 461 different genetic skeletal disease. However, most diseases were not included in ICD-10 code lists and finally a total of 45 ICD-10 codes regarding GSD were recruited (Sup Table 1). Other patients without ICD10 codes related to GSD or rare bone disease were defined as common condition, that is, control population. Due to the difficulty in diagnosing GSD in clinics, we have recruited patients with RBD based on clinical diagnoses including morphological, physiological, and functional abnormalities as a surrogate to identify a study population of patients likely to include most patients with GSD. The RBD was diagnosed based on ICD-10 code list linked to rare bone diseases, or features of them, which was primarily provided by Orphanet [20], a worldwide representative database dedicated to offering information on rare diseases and orphan drugs [21].

### Outcomes

For analyses based on NIS database, the primary outcomes including total charges and length of stay. For NRD database, besides economic cost and hospitalized stay, early all-cause readmission rate within 30 days’ period after first hospitalization were recruited into the final analyses.

### Data collection

Two database contains characteristics related to patient and hospital factors that were collected and analyzed in this study. We extracted patient information including age, sex, race (white, black, Hispanic, Asian or pacific islander, native American, other), expected primary payer (medicare, medicaid, private insurance, self-pay, no charge, other), patient location (“Central” counties of metro areas of > = 1 million population, “Fringe” counties of metro areas of > = 1 million population, Counties in metro areas of 250,000-999,999 population, Counties in metro areas of 50,000-249,999 population, Micropolitan counties, Not metropolitan or micropolitan counties), discharge disposition (Routine, Transfer to short-term hospital, Transfer to other facility, Home health care, Against medical advice, Died in hospital, Discharged/transferred to court/law enforcement, Discharged alive, destination unknown), number of diagnosis and procedure, transferring recording in NIS database. Meanwhile, sample data elements including age, sex, expected primary payer, patient location and median household income for patient’s ZIP code were captured in NRD database.

### Statistical analysis

The baseline characteristics of patients with GSD/RBD and common conditions were outlined. Continuous variables were expressed as mean ± standard error (SE) and compared using an independent two-sample t-test. Categorical variables were presented as proportions and analyzed for between-group differences using the Chi-squared test. The hospitalized outcome including total charges, length of stay and readmission rate were compared between patients with GSD/RBD or common conditions via independent two-sample t-test or Chi-squared test. The readmission rates were presented as percentages from day 1 to 30 after first hospitalization in NRD database. We further selected top six prevalent GSD in two databases and contrast the hospitalized outcomes among these specific diseases. We used SPSS statistical software (version22.0) to perform all statistical analyses and considered a two-sided p value lower than 0.05 as statistically significant.

## Results

### Health-Care utilization in patients with GSD compared to CC

**Fig. 1:**
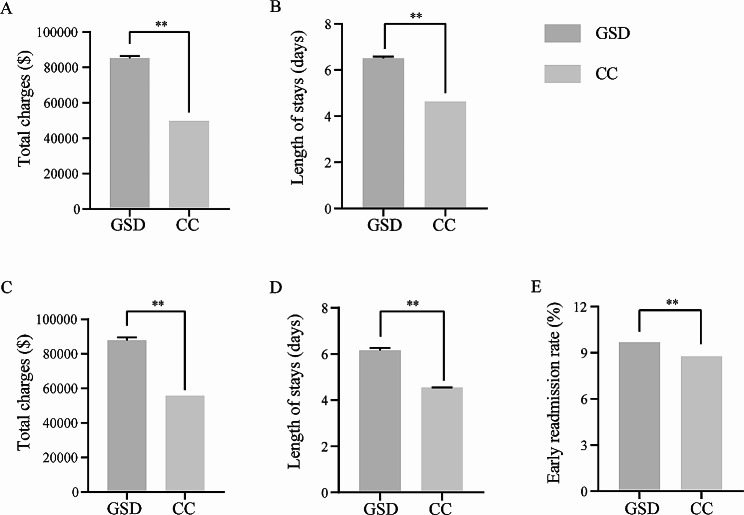
Hospitalized outcomes of inpatient with genetic skeletal disease compared to common conditions based on NIS and NRD database. 1 A: total charges in patients with GSD vs. CC (NIS); 1B: length of stay in patients with GSD vs. CC (NIS); 1 C: total charges in patients with GSD vs. CC (NRD); 1D: length of stay in patients with GSD vs. CC (NRD); 1E: early readmission rate in patients with GSD vs. CC (NRD). **P < 0.01, GSD: genetic skeletal disorders, CC: common conditions

In the study, a total of 25,945 (0.12%) records regarding GSD were extracted from all 21,400,282 records in NIS database. We evaluated health-care utilization, including total charges and length of stay, in hospitalized individuals with GSD. Compared to patients with common conditions, the total charges for patients with GSD increased 1.71 folds (85,180.97 ± 1239.47 vs. 49,884.26 ± 20.99, *P* < 0.001) (Fig. 1A) and the length of stay increased 1.40 folds (6.50 ± 0.08 vs. 4.63 ± 0.002, *P* < 0.001) (Fig. 1B). Our analysis involved a total of 12,928,231 patients, and we identified 14,373 patients diagnosed with GSD based on NRD database. The mean total charges were approximately $31,951 higher for GSD ($87,754.98 ± $1,827.26) than common conditions ($55,803.48 ± $29.00) (Fig. 1C). Patients with GSD experienced a significantly prolonged length of stay than common conditions (6.16 ± 0.11 vs. 4.55 ± 0.002, *P* < 0.001) (Fig. 1D). Meanwhile, data based on NRD showed that patients with GSD had a mild but significantly higher early 30-day all-cause readmission rate than those with common conditions (9.67% vs. 8.77%, *P* < 0.001) (Fig. 1E). These results indicated that patients with GSD had heavier health-care utilization and economical cost than common conditions.

### Baseline characteristics in patients with GSD or CC

Apart from health-care utilization, we compared the demographic characteristics between patients with GSD or CC based on two databases. In contrast to patients with common conditions, patients with GSD exhibited a significantly lower age (31.00 ± 24.72 vs. 49.51 ± 27.38, *P* < 0.001) and had a higher proportion of female (58.40% vs. 56.40%, *P* < 0.001). Furthermore, there were significant differences in the proportion of expected primary payer, patient location, and median household income between patients with GSD and those with common conditions(Table 1). Patients with GSD was more likely to experience procedure (66.80% vs. 61.14%, *P* < 0.001), transferring in (12.37% vs. 8.96%, *P* < 0.001), and possess multiple diagnosis than patients with CC (30.57% vs. 26.81%, *P* < 0.001) (Table 1). It showed a lower proportion of nonelective admission in patients with GSD than common conditions (68.31% vs. 79.13%, *P* < 0.001). Similarly, the distribution of age, sex, patient location, median household income and nonselective admission in patients with GSD in NRD database was roughly consistent with NIS database (Table 2). No significant difference of death rate between patients with GSD and CC in NRD database (*P* = 0.729) (Table 2).

**Table 1:** Baseline characteristics for study population from the NIS

	Genetic skeletal disease	Common condition	P value
N (%)	25,945 (0.12)	21,374,337 (99.88)	
Age (years)	31.00 ± 0.15	49.51 ± 0.01	< 0.001
**Sex (%)**			< 0.001
Female	58.40	56.40	
Male	41.60	43.60	
**Race**			< 0.001
White	72.28	64.95	
Black	9.36	15.24	
Hispanic	12.03	12.53	
Asian or Pacific Islander	2.07	3.11	
Native American	0.57	0.66	
Other	3.70	3.52	
**Payer**			< 0.001
Medicare	24.64	40.42	
Medicaid	30.16	23.00	
Private insurance	39.18	29.45	
Self-pay	2.16	3.98	
No charge	0.11	0.31	
Other	3.74	2.85	
**Location**			< 0.001
“Central” counties of metro areas of > = 1 million population	28.35	30.07	
“Fringe” counties of metro areas of > = 1 million population	26.20	24.02	
Counties in metro areas of 250,000-999,999 population	21.86	20.73	
Counties in metro areas of 50,000-249,999 population	9.39	9.22	
Micropolitan counties	8.24	9.13	
Not metropolitan or micropolitan counties	5.97	6.83	
**Diagnoses per discharge (number)**			< 0.001
0	0.00	0.03	
1–5	18.57	25.90	
6–10	27.14	26.49	
11–15	23.72	20.77	
> 15	30.57	26.81	
**Procedures per discharge (number)**			< 0.001
0	33.20	38.86	
1–5	57.03	55.64	
6–10	7.30	4.56	
11–15	1.96	0.76	
> 15	0.51	0.18	
**Discharge disposition**			< 0.001
Routine	73.09	68.39	
Transfer to short-term hospital Transfer to other facility	2.50	1.97	
Home health care	10.04	14.00	
Against medical advice	11.75	12.37	
Died in hospital	0.87	1.31	
Discharged/transferred to court/law enforcement	1.73	1.94	
Discharged alive, destination unknown	0.02	0.02	
**Elective**			< 0.001
Elective admission	31.69	20.87	
Nonelective admission	68.31	79.13	
**Transfer in**			< 0.001
Not transferred in/newborn	87.63	91.04	
From acute care hospital	9.16	5.97	
From another type of health facility	3.22	3.00	
**Transfer out**			< 0.001
Not a transfer	87.46	84.03	
To acute care hospital	2.50	1.97	
To another type of health facility	10.04	14.00	

P: patients with genetic skeletal disease vs. patients with common condition

**Table 2:** Baseline characteristics for study population from the NRD

	Genetic skeletal disease	Common condition	P value
N (%)	14,373 (0.11)	12,913,858 (99.89)	
Age (years)	36.50 ± 0.20	52.35 ± 0.01	< 0.001
**Sex (%)**			< 0.001
Female	63.09	58.76	
Male	36.91	41.24	
**House income (%)**			< 0.001
0-25th percentile ($1 - $45,999)	21.94	27.32	
26th to 50th percentile ($46,000 - $58,999)	26.51	27.46	
51st to 75th percentile ($59,000 - $78,999)	26.85	24.91	
76th to 100th percentile ($79,000 or more)	24.70	20.30	
**Primary expected payer (%)**			< 0.001
Medicare	28.07	41.36	
Medicaid	25.99	20.80	
Private insurance	40.47	30.72	
Self-pay	2.09	3.68	
No charge	0.22	0.44	
other	3.17	2.99	
**Patient location (%)**			< 0.001
”central” counties of metro areas of ≥ 1 million population	25.41	27.97	
”fringe” counties of metro areas of ≥ 1 million population	27.84	25.61	
counties in metro areas of 250,000-999,999 population	23.65	22.02	
counties in metro areas of 50,000-249,999 population	9.81	9.84	
micropolitan counties	7.61	8.33	
not metropolitan or micropolitan counties	5.69	6.23	
Dead (%)	1.76	1.72	0.729
**Elective**			< 0.001
Elective admission	31.92	23.29	
Nonelective admission	68.08	76.71	

P: patients with genetic skeletal disease vs. patients with common condition

### Subgroup analysis: health-care utilization in patients with different GSD

**Fig. 2:**
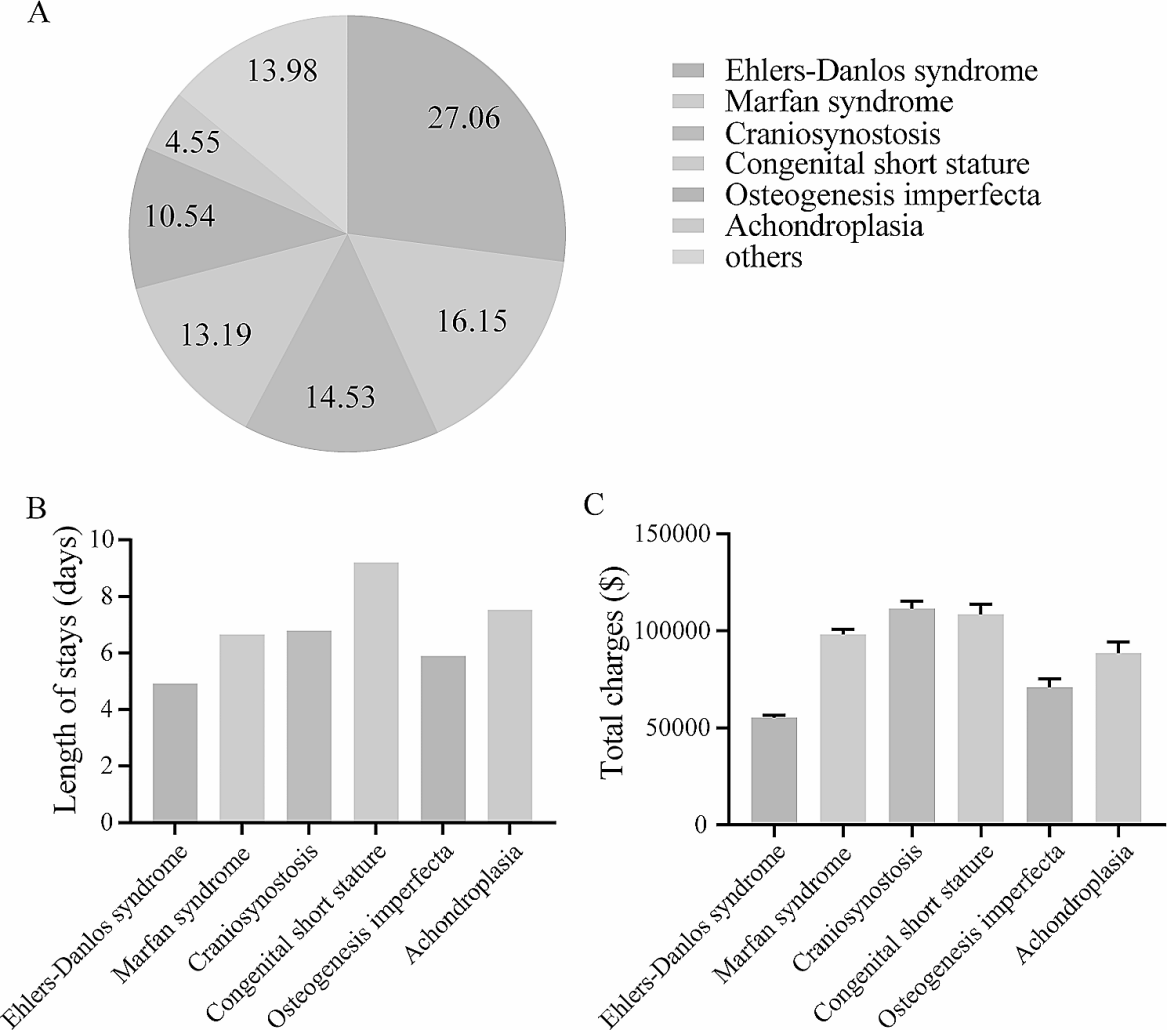
Subgroup analysis: hospitalized outcomes of inpatient with different genetic skeletal disorders in NIS database. 2 A: the proportion of different genetic skeletal disorder in whole population with genetic skeletal disorders; 2B: length of stay in patients with different genetic skeletal disorder; 2 C: total charges in patients with different genetic skeletal disorder

**Fig. 3:**
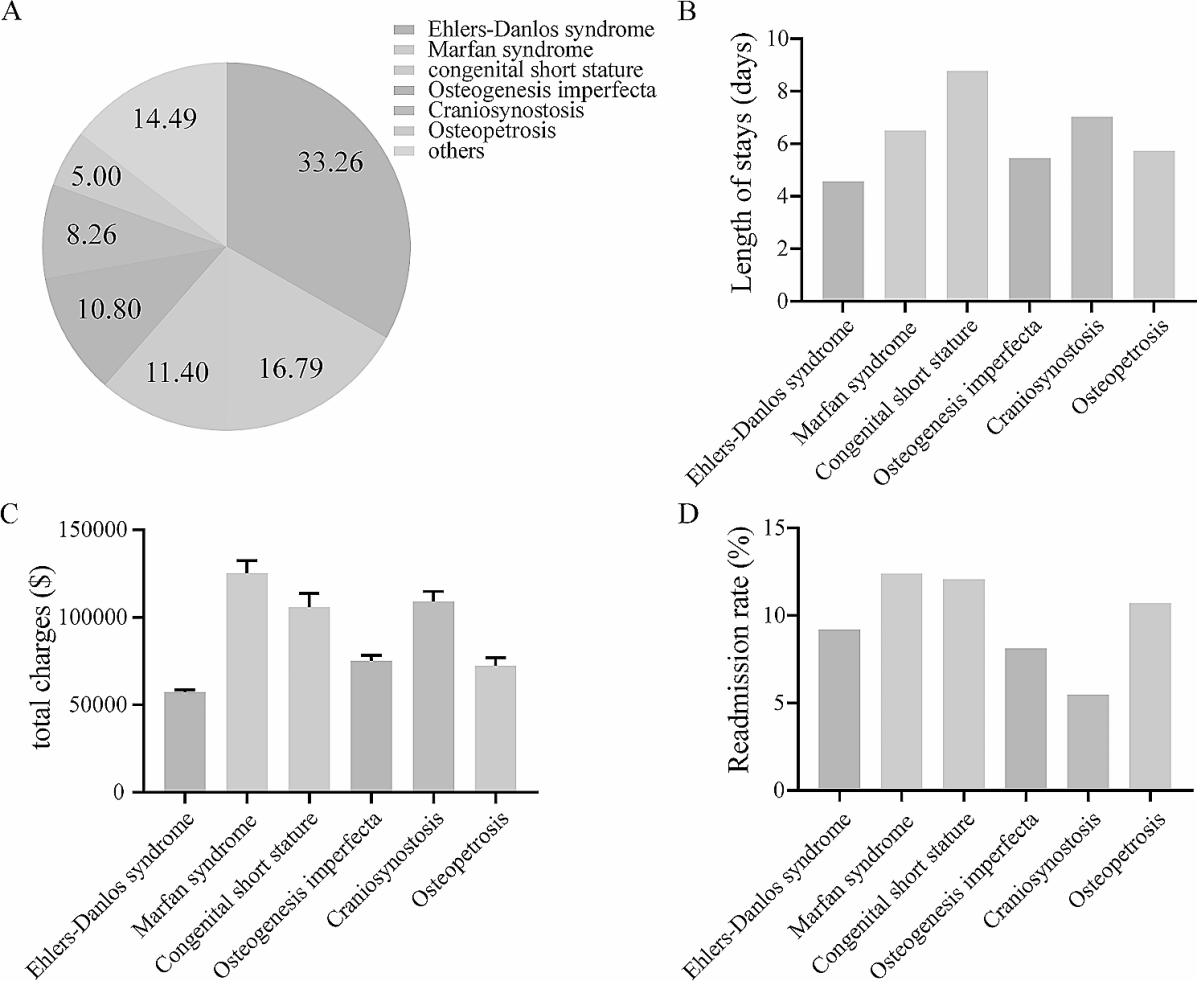
Subgroup analysis: hospitalized outcomes of inpatient with different genetic skeletal disorders in NRD database. 3 A: the proportion of different genetic skeletal disorder in whole population with genetic skeletal disorders; 3B: length of stay in patients with different genetic skeletal disorder; 3 C: total charges in patients with different genetic skeletal disorder; 3D: readmission rate in patients with different genetic skeletal disorder

To further estimate the health-care utilization and economical cost in patients with specific GSD, we computed the percentage of different GSD and performed further analysis in two databases. In NIS database, the most common GSD was Ehlers-Danlos syndrome (27.06%), followed by marfan syndrome (16.15%), craniosynostosis (14.53%), congenital short stature (13.19%), osteogenesis imperfecta (10.54%), achondroplasia (4.55%) and others (13.98%) (Fig. 2A). Correspondingly, the patients with congenital short stature (9.20 ± 0.32) and achondroplasia (7.53 ± 0.41) were likely to have longer hospitalized stay, and the total charges in patients with craniosynostosis ($11,609.04 ± $3,641.66) and congenital short stature ($108,569.91 ± $5,275.84) were higher than $10,000 (Fig. 2B-C). The Ehlers-Danlos syndrome were also the most common GSD in NRD database and accounted for 33.26%, followed by marfan syndrome (16.79%), congenital short stature (11.40%), osteogenesis imperfecta (10.80%), craniosynostosis (8.26%), osteopetrosis (5.00%) and others (14.49%) (Fig. 3A). And the mean hospitalized stay of length in patients with congenital short stature (8.77 ± 0.53) and craniosynostosis (7.02 ± 0.52) were over 7 days (Fig. 3B). In addition, the top two highest mean hospitalized cost were $125,236.24 for marfan syndrome and $109,025.37 for craniosynostosis respectively (Fig. 3C). The early 30-day all-cause readmission rate in patients with marfan syndrome (12.39%), congenital short stature (12.08%) or osteopetrosis (10.71%) were higher than 10% (Fig. 3D). The detailed results about hospitalized outcomes in above mentioned subgroups were shown in Fig. 2 and 3.

### Health-Care utilization in patients with RBD compared to CC

**Fig. 4:**
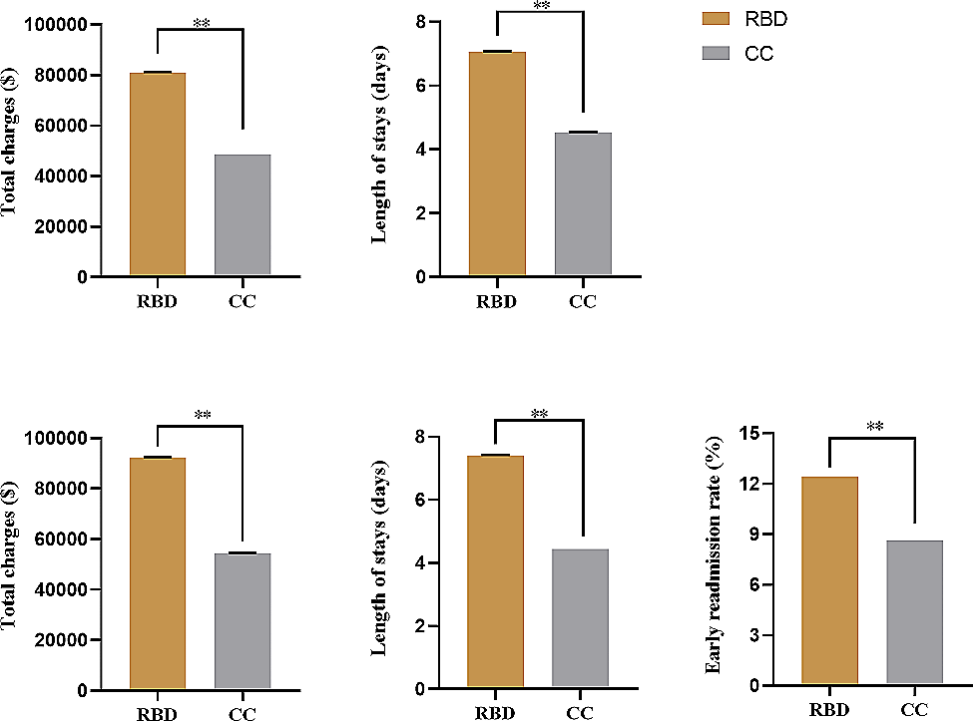
Hospitalized outcomes of inpatient with rare bone disease compared to common conditions based on NIS and NRD database. 4 A: total charges in patients with RBD vs. CC (NIS); 4B: length of stay in patients with RBD vs. CC (NIS); 4 C: total charges in patients with RBD vs. CC (NRD); 4D: length of stay in patients with RBD vs. CC (NRD); 4E: early readmission rate in patients with RBD vs. CC (NRD). **P < 0.01, RBD: rare bone disease, CC: common conditions

Given the challenges in diagnosing GSD in clinical settings and imperfections in coding system, we identified patients with rare bone disease via ICD-10 code list associated with rare bone diseases, or features of them in two databases. We identified 797,916 (3.73%) patients with RBD from NIS database. Similarly, the patients with RBD exhibited a significantly longer hospitalized stay of length (7.05 ± 0.01 vs. 4.54 ± 0.001, *P* < 0.001) (Fig. 4A) and higher total charges ($80,809.75 ± 176.53 vs. $48,734.71 ± 20.69, *P* < 0.001) (Fig. 4B) in comparison to patients with common conditions. And a total of 464,870 patients (3.60%) with RBD were identified in NRD database. The length of stay increased roughly 1.67-fold (7.40 ± 0.02 vs. 4.44 ± 0.002, *P* < 0.001) and the mean total charges were approximately $37,850 higher ($92,327.96 ± 241.23 vs. $54,477.88 ± 28.67, *P* < 0.001) for patients with GSD than those with common conditions (Fig. 4B-C).Additionally, patients with RBD showed a higher early all-cause readmission rate (12.42% vs. 8.63%, *P* < 0.001) compared to patients with common conditions (Fig. 4D). The detailed demographic characteristics between patients with RBD or CC based on NIS and NRD databases were showed in supplement Tables 2 and 3.

## Discussion

It is a critical public health issue to assess the health-care utilization and economic cost in patients with GSD due to its complexity and high deformity and disability although it is extremely rare. A systematic evaluation of hospitalized recordings is essential to draw attention to GSD at both the systemic and individual levels within the healthcare system. In this study, we collected 21,400,282 and 12,928,231 inpatients records in NIS and NRD database respectively, distinguished patients with GSD and performed further analysis. The results showed that patients with GSD exhibited significantly higher length of stay, total charges, early all-cause readmission, and a great number of procedure, diagnosis and transferring records in comparison to patients with CC. Therefore, it is needed and urgent to pay attention to patients with GSD due to its heavy health-care utilization and economical cost.

To the best of knowledge, it was the first study to comprehensively test health-care utilization and economical cost in patients with GSD. Previous studies have explored the health-care utilization and economic burden in patients with rare disease or pediatric genetic disease in United States [1–2]. As expected, the results showed that patients with rare disease or pediatric genetic disease experienced a significantly heavier health-care utilization and financial cost compared to patients with common conditions. However, it is noteworthy that the mean charges in patients with GSD in our study were apparently higher than those reported for patients with rare disease in previous reported study based on NIS ($85,181 vs. $69,275) and NRD ($87,755 vs. $66,675) database [1]. And there was a slight increase in the mean hospitalized length of stay for patients with GSD compared to individuals with total rare diseases, as evidenced by the comparison between our data and previous results. The heavier burden in rare bone disorders emphasized the need for delivering high-quality health care for patients affected by this specific rare disease. Another recent study has pointed that hospital inpatient care and prescription medication were the top direct cost categories for economical costs associated with rare disease, labor market productivity losses were the top indirect cost categories [22]. Consequently, it is essential to conduct additional research to comprehensively evaluate the overall economic burden in patients with GSD, considering both direct medical costs and indirect costs.

The results showed that patients with GSD possessed significantly more items of diagnosis, undertaking higher number of procedures, and suffering increased hospital transferring. It indicated that the increased prevalence of concomitant complication, the necessary of operative treatment and the frequent hospital transferring, all stemming from the complexity and severity of GSD, contribute significantly to the high economic burden in patients with GSD. It has been demonstrated that diagnostic delays of rare disease contribute to the financial burden [23–24]. Although the records of diagnostic course for each patient could not be obtained via the present database, the longer hospitalized stay serves as an indicator of the challenges associated with receiving a definitive diagnosis for patients with GSD. Meanwhile, the early all-cause readmission rate in patients with GSD was significantly higher than patients with common conditions. It has been reported that the cost of unplanned readmission was up to $17 billion [25]. Minimizing hospital readmissions is an increasingly prioritized goal within the healthcare system due to its significant economic burden and adverse outcomes.

Given the limitations inherent in gene sequencing availability in clinical settings and the relatively low detection sensitivity of ICD-10 codes, it is inevitable that the prevalence and economic burden in this study may be somewhat underestimated. Nevertheless, the results for examination of health-care utilization and economic burden were alarming. Meanwhile, in order to increase the detection sensitivity, we distinguish patients with rare bone disease based on ICD-10 codes linked to rare bone disease or features of them, as provided by Orphanet [20–21]. Based on this classification, some patients may not undertake genetic sequencing or may simply have similar symptoms of a rare disease. While this approach maximizes the inclusion of patients with underlying genetic skeletal disorders, it is important to note that, despite the rarity of GSD, certain relatively common GSDs merit attention. It showed that Ehlers-Danlos syndrome was the most common GSD in two databases, which was consistent with previous study. It has been reported that the hypermobile type of Ehlers–Danlos syndrome was likely the most common systemic inherited connective tissue disorder [26]. While patients with congenital short stature, craniosynostosis or marfan syndrome were likely to have relatively higher total charges and longer hospitalized stay. Hence, the results underscore the immediate need to enhance the care, management, and treatment of these particular diseases.

It was the first study to comprehensively assess the health-care utilization and economic burden in patients with GSD. The results underscored the substantial economic burden in patients with GSD and arouse the attention for these diseases. However, several limitations existed in this study. The reliance on ICD-10 codes may result in the inaccuracy of diagnosis, given that ICD-10 codes have not yet been specifically validated for the identification of genetic skeletal disorders. Meanwhile, the number of GSD included in this analysis was far from the 461 genetic skeletal disorders recently described by Nosology. To avoid underestimating the prevalence and economic burden, we extracted patients with rare bone disease via ICD-10 code list linked to rare bone diseases, or features of them to maximize the inclusion of patients. And it was also inevitable that some patients with GSD were undiagnosed or diagnosed incorrectly due to the imperfection of ICD10 code and lack of information about past hospitalizations, which was not included in the GSD cohort. However, the underestimation of prevalence and burden further aroused the attention of public to this population.

## Conclusions

In conclusion, we found that patients with GSD were likely to have significantly higher length of stay, total charges, early all-cause readmission, suffering more procedure, diagnosis and transferring records in comparison to patients with CC. The substantial health-care utilization and economic burden underscore the urgency for policymakers, scientific and pharmaceutical researchers, healthcare providers, and employers to promptly identify innovative strategies and implement effective measures. This concerted effort is crucial to enhance the care, management, and treatment of these debilitating diseases.

### Electronic supplementary material

Below is the link to the electronic supplementary material.

Supplementary Material 1

## Data Availability

The data used for study were obtained from Nationwide Inpatient Sample and Nationwide Readmission Database.
