# Supplementary material for: The health-care utilization and economic burden in patients with genetic skeletal disorders
Source: Orphanet J Rare Dis. 2024 Mar 4;19:99. doi: 10.1186/s13023-024-03102-3 (PMC10913423; doi:10.1186/s13023-024-03102-3)
Supplement: Supplementary file 1 — Supplementary Material 1 [file 13023_2024_3102_MOESM1_ESM.docx]

Supplement Table 1 List of genetic skeletal disease recruited in the analysis.

| Name of disorder | OMIM number | ORPHANET number | ICD_10 codes |
| --- | --- | --- | --- |
| Achondrogenesis (type 1A, 1B, 2) | 200600,200610, 600972 | 93296, 93298, 93299 | Q770 |
| Achondroplasia | 100800 | 15 | Q774 |
| Thanatophoric dysplasia (type 1, 2) | 187600,187601 | 18060,93274 | Q771 |
| Spondyloepiphyseal dysplasia | 183900, 616583, 604864, 184250, 184253, 184255 | 94068, 93346, 93316, 93315, 85198 | Q777 |
| Diastrophic dysplasia | 222600 | 628 | Q775 |
| Ehlers-Danlos syndrome | 601776, 615539, 612350, 225400, 614557, 130070 | 2953, 157965, 1900, 300179, 75497 | Q796, Q7960, Q7961, Q7962, Q7963, Q7969 |
| Myotonic chondrodystrophy | 255800 | 800 | G7113 |
| Chondroectodermal dysplasia | 225500 | 289 | Q776 |
| Metaphyseal dysplasia | 156500, 250250, 617396, 156400, 600002, 260400, 617941, 602111, 613073, 250400, 156510, 605946 | 174, 175, 93347, 33067, 79106, 811, 1040, 2501, 2504, 85188 | Q785 |
| congenital malformation syndromes predominantly associated with short stature | 617662, 614800 | 527450, 391677 | Q871, Q8719 |
| Chondrodysplasia punctata | 302960, 302950, 118651, 215100, 222765, 600121, 616154, 616716 | 35173, 79345, 79346, 177 | Q773 |
| Rhizomelic chondrodysplasia punctata | 215100, 222765, 600121, 616154, 616716 | 177 | E71540 |
| Osteopetrosis | 259700, 611490, 615085, 259720, 612301, 259710, 611497, 259710, 259730, 166600, 300301, 612840 | 667, 85179, 178389, 210110, 2785, 53, 69088, 99844 | Q782 |
| Progressive diaphyseal dysplasia | 131300 | 1328 | Q783 |
| Osteogenesis imperfecta | 166200, 166210, 610854, 610915, 259440, 259420, 610967, 613982, 610682, 613848, 610968, 615066, 112264, 615220, 616229, 616507, 617952, 166220, 613849, | 216796, 216804, 216812, 216820, 216828 | Q780 |
| Hypophosphatemic rickets | 241500, 146300, 307800, 193100, 241520, 613312, 300554, 241530 | 436, 247676, 89936, 89937, 289176, 1652, 157215 | E8331 |
| Vitamin D-dependent rickets | 264700, 600081, 277440, 600785 | 289157, 93160 | E8332 |
| Mucopolysaccharidosis | 607014, 607015, 607016, 309900, 252900, 252920, 252930, 252940, 253000, 253010, 253200, 253220, 617303 | 579, 580, 79269, 79270, 79271, 79272, 309297, 309310, 583, 584, 505248 | E761, E76210, E76211, E76219, E7622, E7629, E763 |
| GM1 Gangliosidosis | 230500 | 354 | E7509, E7519 |
| Mucolipidosis | 252500, 252600, 252605 | 576, 423461, 423470 | E770 |
| Polyostotic fibrous dysplasia | 174800 | 562 | Q781 |
| Enchondromatosis | 166000 | 296, 163634 | Q784 |
| Marfan syndrome | 154700 | 558 | Q874, Q87410, Q87418, Q8742, Q8743 |
| Craniosynostosis | 123790, 602849, 604757, 615314, 600775 | 1555, 53271, 1541, 35099 | Q750 |
| Dysostoses with predominant craniofacial involvement | 154500, 248390, 613717, 610536, 616367, 263750, 154400, 201170, 616462, 136760, 613451, 613456, 304110, 603671, 164210, 268305, 602483, 614669, 615706, 311200, 193530 | 861, 79113, 443995, 246, 245, 1788, 1200, 391474, 228390, 306542, 1520, 1827, 374, 3102, 137888, 2750, 952 | Q751, Q754, Q755 |
| Pseudohypoparathyroidism | 103580 | 79443 | E201 |

Supplement Table 2 Baseline characteristics for study population from the NIS.

|  | Rare bone disease | Common condition | P value |
| --- | --- | --- | --- |
| N (%) | 800820 (3.74) | 20599462 (96.26) |  |
| Age (years) | 53.34±0.03 | 49.33±0.01 | <0.001 |
| Sex (%) |  |  | <0.001 |
| female | 56.08 | 56.42 |  |
| male | 43.92 | 43.58 |  |
| Race |  |  | <0.001 |
| White | 62.68 | 65.04 |  |
| Black | 17.55 | 15.14 |  |
| Hispanic | 11.90 | 12.55 |  |
| Asian or Pacific Islander | 3.54 | 3.09 |  |
| Native American | 0.70 | 0.65 |  |
| Other | 3.63 | 3.52 |  |
| Payer |  |  | <0.001 |
| Medicare | 49.88 | 40.03 |  |
| Medicaid | 20.34 | 23.11 |  |
| Private insurance | 23.91 | 29.68 |  |
| Self-pay | 3.26 | 4.00 |  |
| No charge | 0.25 | 0.31 |  |
| Other | 2.36 | 2.87 |  |
| Location |  |  | <0.001 |
| "Central" counties of metro areas of >=1 million population | 31.76 | 30.00 |  |
| "Fringe" counties of metro areas of >=1 million population | 24.78 | 23.99 |  |
| Counties in metro areas of 250,000-999,999 population | 20.49 | 20.74 |  |
| Counties in metro areas of 50,000-249,999 population | 9.11 | 9.23 |  |
| Micropolitan counties | 8.00 | 9.17 |  |
| Not metropolitan or micropolitan counties | 5.86 | 6.86 |  |
| Diagnoses per discharge (number) |  |  | <0.001 |
| 0 | 0.00 | 0.03 |  |
| 1-5 | 11.06 | 26.47 |  |
| 6-10 | 15.67 | 26.91 |  |
| 11-15 | 20.71 | 20.77 |  |
| >15 | 52.56 | 25.82 |  |
| Procedures per discharge (number) |  |  | <0.001 |
| 0 | 33.73 | 39.05 |  |
| 1-5 | 55.26 | 55.65 |  |
| 6-10 | 8.28 | 4.42 |  |
| 11-15 | 2.12 | 0.71 |  |
| >15 | 0.62 | 0.17 |  |
| Discharge disposition |  |  | <0.001 |
| Routine | 55.03 | 68.91 |  |
| Transfer to short-term hospital  Transfer to other facility | 2.22 | 1.96 |  |
| Home health care | 21.85 | 13.69 |  |
| Against medical advice | 16.32 | 12.21 |  |
| Died in hospital | 1.09 | 1.32 |  |
| Discharged/transferred to court/law enforcement | 3.46 | 1.88 |  |
| Discharged alive, destination unknown | 0.01 | 0.02 |  |
| Elective |  |  | <0.001 |
| Elective admission | 16.15 | 21.07 |  |
| Nonelective admission | 83.85 | 78.93 |  |
| Transfer in |  |  | <0.001 |
| Not transferred in/newborn | 89.34 | 91.10 |  |
| From acute care hospital | 7.27 | 5.92 |  |
| From another type of health facility | 3.39 | 2.98 |  |
| Transfer out |  |  | <0.001 |
| Not a transfer | 75.92 | 84.35 |  |
| To acute care hospital | 2.22 | 1.96 |  |
| To another type of health facility | 21.85 | 13.69 |  |

Supplement Table 3 Baseline characteristics for study population from the NRD.

|  | Rare bone disease | Common condition | P value |
| --- | --- | --- | --- |
| N (%) | 464870 (3.60) | 12463361 (96.40) |  |
| Age (years) | 58.47±0.04 | 52.10±0.01 | <0.001 |
| Sex (%) |  |  | <0.001 |
| female | 57.75 | 58.81 |  |
| male | 42.25 | 41.19 |  |
| House income (%) |  |  | <0.001 |
| 0-25th percentile ($1 - $45,999) | 25.71 | 27.38 |  |
| 26th to 50th percentile ($46,000 - $58,999) | 26.20 | 27.51 |  |
| 51st to 75th percentile ($59,000 - $78,999) | 25.57 | 24.89 |  |
| 76th to 100th percentile ($79,000 or more) | 22.52 | 20.23 |  |
| Primary expected payer (%) |  |  | <0.001 |
| Medicare | 54.71 | 40.85 |  |
| Medicaid | 16.60 | 20.97 |  |
| Private insurance | 22.99 | 31.02 |  |
| Self-pay | 2.95 | 3.71 |  |
| No charge | 0.35 | 0.44 |  |
| other | 2.40 | 3.02 |  |
| Patient location (%) |  |  | <0.001 |
| ”central” counties of metro areas of ≥1 million population | 29.50 | 27.91 |  |
| ”fringe” counties of metro areas of ≥1 million population | 27.39 | 25.54 |  |
| counties in metro areas of 250,000-999,999 population | 21.19 | 22.06 |  |
| counties in metro areas of 50,000-249,999 population | 9.57 | 9.85 |  |
| micropolitan counties | 7.05 | 8.38 |  |
| not metropolitan or micropolitan counties | 5.31 | 6.27 |  |
| Dead (%) | 3.56 | 1.65 | <0.001 |
| Elective |  |  | <0.001 |
| Elective admission | 16.83 | 23.54 |  |
| Nonelective admission | 83.17 | 76.46 |  |
